# Thermophysical Properties and Molecular Dynamics Insights into Glycolic Acid–Sorbitol NaDES–Water Mixtures

**DOI:** 10.1021/acsomega.6c00721

**Published:** 2026-05-02

**Authors:** Marcelo M. do Ó, Sádwa F. Ribeiro, Lucas L. Bezerra, Norberto de K. V. Monteiro, Alanderson A. A Alves, Francisco H. B. Quinto, Rilvia S. de Santiago-Aguiar

**Affiliations:** † Department of Chemical Engineering, 28121 Federal University of Ceará, Pici Campus, Bloco 731B, 60440-900 Fortaleza, CE, Brazil; ‡ Department of Analytical and Physical Chemistry, Federal University of Ceará, Pici Campus, Bloco 938/939, 60440-900 Fortaleza, CE, Brazil; § 74384 Federal Rural University of the Semi-Arid Region, 59625-900 Mossoró, Rio Grande do Norte, Brazil

## Abstract

Natural deep eutectic solvents (NaDES) have emerged as a sustainable alternative to traditional organic solvents due to their easy preparation, biodegradability, low toxicity, and high chemical stability. NaDESs are formed and their physicochemical properties can be tailored by solvent addition and water content. In this study, density, viscosity, and electrical conductivity of binary mixtures composed of glycolic acid and sorbitol (3:1) NaDES and water were investigated. The results show a reduction in density and viscosity with increasing temperature and water mole fraction, while conductivity increases up to a maximum near a water mole fraction of 0.9 and decreases in the water-rich region. Excess molar volumes and viscosity deviations were calculated and correlated using the Redlich–Kister equation. FTIR spectroscopy and molecular dynamics simulations revealed that hydrogen-bond interactions between NaDES components and water are progressively weakened with increasing water content, explaining the observed changes in transport properties.

## Graphical abstract

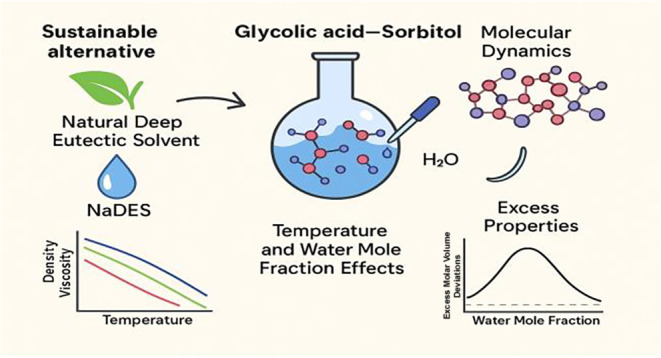

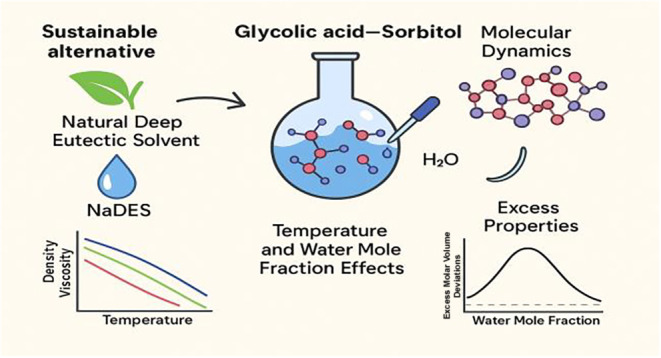

## 1 Introduction

Deep eutectic solvents (DESs) are recognized as a class of green solvents analogous to ionic liquids (ILs), as they share some general characteristics, including low volatility, low vapor pressures, thermal stability, and adjustable polarity.^1,2^ However, DESs have a great advantage over ionic liquids as they present greater biodegradability, lower toxicity, lower cost and easier synthesis, consisting of a simple mixture between a hydrogen bond acceptor (HBA) and a hydrogen bond donor (HBD) at moderate temperatures.^3–5^

Although DESs offer improvements over ionic liquids, the growing concern about environmental and safety issues has encouraged the search for more natural components for the synthesis of biocompatible solvents, such as sugars, organic acids, amino acids, and organic bases, leading to natural deep eutectic solvents (NaDESs).^6–8^ NaDESs have been applied in the most diverse fields, such as gas absorption,^9^ biocompound extraction,^10^ biomass pretreatment,^11^ biocatalysis,^12^ drug delivery systems,^13^ among others.

These applications are strongly influenced by the physicochemical properties of deep eutectic solvents, such as density, viscosity, conductivity, polarity, and surface tension, among others, which are largely determined by intermolecular hydrogen bond interactions.^14,15^ In this sense, the optimization of DESs properties has been studied to improve them and make them more suitable for use under different operating conditions.^16^

One way to modulate these properties is by adding other solvents, highlighting water as a favorable option because it is environmentally safe, has low viscosity and has a great influence on altering the physical-chemical properties of DESs even at low concentrations.^17,18^ In this context, the density of different molar fractions of deep eutectic solvents and water has been evaluated in several studies, along with the excess molar volume, a fundamental parameter for understanding the behavior of DESs in the presence of other solvents.^19,20^ This is because this property depends on molecular organization and is affected by the existence of holes and vacancies within deep eutectic solvents.^15^

Furthermore, the viscosity of binary mixtures of DES and water has attracted interest, as this parameter plays a determining role in operations that include fluid flow and heat and mass transfer.^21^ Viscosity is strongly influenced by the strengths of the system’s hydrogen bonds, therefore, it is important to evaluate the role of water in establishing these interactions, Gajardo-Parra et al.^22^ and Ghaedi et al.,^23^ for example, investigated the effects of water molar fraction and temperature on the viscosity of a DES based on phosphonium and diethylene glycol and observed that the properties deviate from their ideal behavior, which can be understood as excess thermodynamic properties.

Conductivity is also an essential parameter in characterizing the structure and aggregation behavior of deep eutectic solvents,^24^ as many applications are limited by conductivity in the electrochemical industry Boublia et al.^25^ and Wang et al.^16^ found that the conductivity of DES binary systems of glycolic acid and xylitol with water was related to viscosity and gradually increased with increasing water molar fraction and temperature.

Computational approaches can be successfully applied to NaDESs, providing important tools for predicting properties and understanding the predominant interactions.^26^ Classical molecular dynamics has been successfully applied to various types of deep eutectic solvents. It was used by Sun et al.^27^ to analyze different mixtures of choline chloride and urea, providing possible explanations for the low melting point of the eutectic mixture, and has been applied in several studies of the interactions in different DESs.^28–32^ The influence of water content on the composition of DESs has also been analyzed through molecular dynamics in several studies.^33–37^

In this sense, the present study determined the density, viscosity and conductivity of the binary mixture composed of the natural deep eutectic solvent of glycolic acid and sorbitol (3:1) and water at atmospheric pressure and at different temperatures *T* = (303.15 K to 343, 15 K) with the aim of obtaining data that can contribute to the design of DESs with optimized properties, furthermore, the molecular dynamics was applied to analyze the interactions between NaDES and water.

## 2 Materials and Methods

The chemicals employed in this study are summarized in Table 1. All reagents were used as received from the suppliers without further purification. D-sorbitol and glycolic acid of analytical grade were used in the preparation of the mixtures. Deionized water was employed in order to minimize the presence of ions or impurities that could affect the measured properties of the systems studied.

**Table 1:**
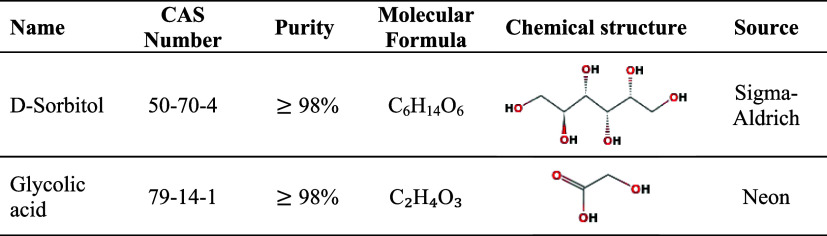
Specifications and Sources of the Chemicals Used in This Work

NaDES was prepared according to an adaptation of the methodology developed by Abbott et al.^38^ Glycolic acid (HBD) and sorbitol (HBA) were weighed on an analytical balance (Shimadzu ATX224) and mixed at a 3:1 molar ratio, respectively. This was the optimal molar ratio attributed to its lowest melting point for a NaDES of glycolic acid (HBD) and xylitol (HBA), according to Liu and Tian.^39^ The synthesis occurred under heating in a water bath, with a temperature of 363.15 K, and constantly stirring for 30 min until a homogeneous liquid was obtained. The prepared NaDES was stored in a vacuum desiccator for later use.

### 2.1 Characterization of NaDES

Water content was measured using a Karl Fischer (Metrohm 870 KF Titrino Plus). Density and viscosity were determined using a digital viscometer (SVM 3000—Anton Paar) with an uncertainties of 0.35% for viscosity and 0.0005 g/cm^3^ for density. Conductivity was measured on a bench conductivity meter (DS-703A—Satra) with an accuracy of ± 0.5% FS. The reported uncertainties correspond to combined uncertainties, taking into account the instrumental accuracy as well as contributions from weighing procedures and the purity of the chemicals used in the preparation of the mixtures. FTIR analysis was performed on a Fourier transform infrared spectrometer using the Cary 630 device from Agilent Technologies. Transmission spectra were collected in the wavenumber range of 600–4000 cm^–1^ with a spectral resolution of 1 cm^–1^ and 32 scans.

The structure of the NaDES was also characterized by NMR spectra. They were obtained on an Agilent DD2 600 MHz instrument (for ^1^H nucleus) and equipped with a One Probe with 5 mm internal diameter (HF/15N-31P) for inverse detection and field gradient on the “*z*” axis. The samples were prepared by mixing 50 μL of sample with 550 μL of a stock solution of deuterated dimethyl sulfoxide (DMSO-*d*_6_, Cambridge Isotope Laboratories). To perform the ^1^H NMR experiment, a waiting time between each acquisition of 2 s, AQ of 3.3 s, gain of 26, 32 transients in a spectral window of 16 ppm and 32k number of real points at 26 °C were used. For data processing, the spectra were referenced with the internal standard tetramethylsilyl propionate (TMSP-d4) at 0 ppm. For ^13^C, a waiting time between each acquisition of 1 s, AQ of 0.8 s, 2k transients in a spectral window of 251 ppm and 32k number of points were used. The data obtained from the characterization of NaDES are available in Supporting Information.

#### 2.1.1 NaDES NMR

To confirm the synthesis of NaDES (Glycolic acid (HBD) and sorbitol (HBA)), the structure of the NaDES was also characterized by ^1^H NMR and ^13^C NMR. (Figures S1 and S2 in Supporting Information).

Figure S1a shows peaks in (1) δ­(ppm) = 4.55 (SOR OH), (2) δ­(ppm) = 3.60 (SOR CH_2_), (4) δ­(ppm) = 3.99 (SOR OH), (14) δ­(ppm) = 3.87 (GA CH2) e (15) δ­(ppm) = 4.05 (GA OH). In the Figure S1b, the signs were observed in (1) δ­(ppm) = 4.55­(SOR OH), (2) δ­(ppm) = 3.50 (SOR CH_2_), (3) δ­(ppm) = 3.63 (SOR CH), (4) δ­(ppm) = 3.99 (SOR OH), (5) δ­(ppm) = 3.67 (SOR CH), (6) δ­(ppm) = 3.73 (SOR OH), (14) δ­(ppm) = 3.87 (GA CH_2_) e (15) δ­(ppm) = 4.05 (GA OH).

The deep eutectic solvent (DES) formed with glycolic acid and sorbitol is stabilized by strong intermolecular interactions, mainly hydrogen bonds, which can slightly modify the peaks positions relative to the pure compounds. This is because the bond to hydroxyl groups (−OH) or ester groups, such as that of glycolic acid, can cause a deshielding of the protons on the carbon atoms, moving the signals. Thus, the protons on the C4, C5, and C6 carbons of sorbitol, which are directly bonded to oxygen atoms, can generate slightly deshielded signals in this region, moving the signals to the range around 4.0 and 4.8 ppm.

The multiple signals represented by peaks 2,3,5 correspond to the aliphatic protons on the CH and CH_2_ groups of sorbitol, which are more complex due to the presence of several hydroxyl groups (−OH) in different positions. In this sense, the multiplicity of these peaks can change due to the possible hydrogen bonds established with glycolic acid.

The peak at 3.87 ppm, corresponding to the methylene group (−CH_2_) attached to the carboxylic group (C=O) in glycolic acid, is the strongest peak in the spectrum, indicating a significant amount of glycolic acid in the sample. Furthermore, the relative intensity between the peaks can help confirm the system ratio (3:1 GA). The broad peak around 3.50 ppm in the spectrum is quite characteristic of systems containing polyalcohols such as sorbitol. It can be attributed to the superposition of signals from multiple aliphatic protons (CH and CH_2_) of the six carbon atoms of sorbitol attached to hydroxyl groups (−OH), each with slight variations in the chemical environment. Furthermore, protons from hydroxyl groups (−OH), if not completely exchanged with the solvent (DMSO), can contribute to the coalescence and broadening of the signal. However, in many cases with DMSO, these peaks are strongly attenuated or even invisible, which explains why some hydroxyl protons could not be observed in the spectrum.

The ^13^C NMR chemical shifts of NaDES are shown in Figure S2. The chemical shifts of each carbon in the molecules were confirmed, indicating that the NaDES molecules had been successfully synthesized. Then, according to the 1H NMR, 13C NMR and FTIR analyses, it was possible to verify the synthesis of NaDES (Glycolic acid (HBD) and sorbitol (HBA)).

### 2.2 Characterization of Binary Mixtures

Characterization of NaDES-water binary mixtures was performed at atmospheric pressure with a temperature control accuracy of ±0.1 K, including density, viscosity and conductivity. Density and viscosity were determined using a digital viscometer (SVM 3000—Anton Paar) with an uncertainty of 0.35% for viscosity and 0.0005 g/cm^3^ for density. Conductivity was measured on a bench conductivity meter (DS-703A—Satra) with an accuracy of ± 0.5% FS. FTIR analysis was performed on a Fourier transform infrared spectrometer using the Cary 630 device from Agilent Technologies. Transmission spectra were collected in the wavenumber range of 600–4000 cm^–1^ with a spectral resolution of 1 cm^–1^ and 32 scans.

### 2.3 Computational Details

The tridimensional structure of sorbitol (SOR), glycolic acid (GLY), and water (WAT) molecules was optimized in the gas phase based on the Density Functional Theory (DFT) calculations, through B3LYP hybrid functional^40–42^ and 6–31g­(d,p)^43^ basis-set using the Gaussian09 software.^44^ The absence of negative frequencies was observed in all optimized structures analyzed. These structures were used as a starting point for molecular dynamics (MD) simulations.

All the MD simulations were performed using the GROningen MAchine for Chemical Simulations (GROMACS) 2023.2 version software.^45^ The NaDESs (*x*_1_ = 0.0), NaDESs-Water (*x*_1_ = 0.0052), and Water (*x*_1_ = 1.0) systems were simulated at three temperatures of 303.15, 323.15, and 343.15 K. The mole fraction of water used in the simulations (*x*_1_ = 0.0052) corresponds to the molecular composition of the simulation box described in Table S1 and does not represent the experimental bulk composition. These systems were simulated in a cubic box with initial dimensions of 13 nm × 13 nm × 13 nm. The *x*_1_ is based on the experimental section, and the number of components in each system is present in Table S1 (Supporting Information). These systems were described through the OPLS-AA force field.^46^ Furthermore, the water molecules were described using the SPC/E model.^47^ Figure 1 shows the optimized tridimensional structures employed in the MD simulations.

**Figure 1:**
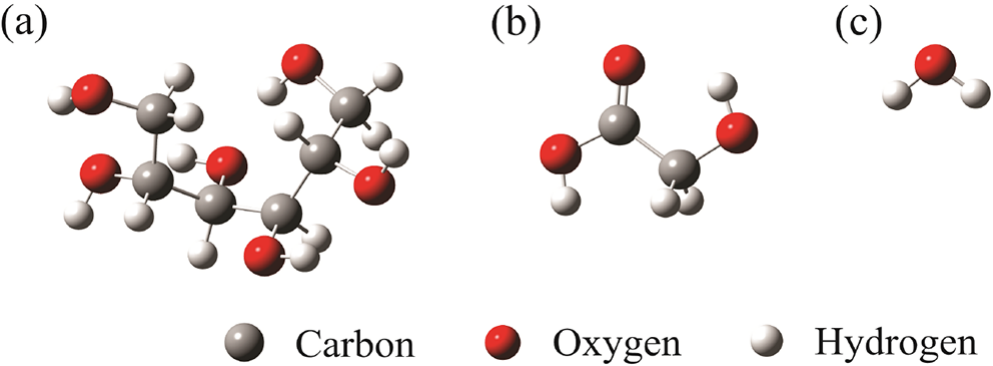
Optimized tridimensional structures of (a) SOR, (b) GLY, and (c) WAT.

The geometry of the system was optimized using the steepest descent^48^ algorithm, followed by the conjugate gradient,^49^ both algorithms with a tolerance energy of 1 kJ mol^–1^ nm^–1^. Posteriorly, the systems were equilibrated using an NVT ensemble with the V-rescale thermostat^50^ to achieve temperatures of 303.15, 323.15, and 343.15 K for the respective systems, followed by the NPT ensemble with the C-rescale barostat^51^ to reach a pressure of 1.0 bar. The NVT and NPT ensembles were performed in 1 and 5 ns, respectively. Finally, the production step was performed for 120 ns using NVT and NPT ensembles through the Leap-Frog integrator.^52^ Furthermore, the same temperatures utilized in the dynamic equilibrium were also simulated in the production step. To validate the MD simulations, eq 1 was utilized to calculate the percentual error between the density values obtained from experimental systems (ρ_exp_) and MD simulations (ρ_sim_).

1 $$\mathrm{error}=\frac{\left|\rho_{\exp}-\rho_{\mathrm{sim}}\right|}{\rho_{\exp}}\times 100\%$$

To measure the affinity between the components, present in these systems, the Interaction Potential Energy (IPE) is calculated by the sum of electrostatic (*V*_elec_) and van der Waals (*V*_vdW_) short-range energies present in eq 2.^53^ Furthermore, *N_i_* and *N_j_* are associated with the total number of atoms *i* and *j*, respectively.

2 $${\mathrm{IPE}}_{i,j}=\sum_{i}^{N_{i}}V_{\mathrm{elec}}\left(r_{\mathrm{ij}}\right)+\sum_{j\neq i}^{N_{j}}V_{\mathrm{vdW}}{(r}_{\mathrm{ij}})$$

## 3 Results and Discussion

The water content of NaDES was determined by the Karl Fischer method, performed in duplicate, and found a value of 0.84%. Due to this low value, the wastewater was considered negligible, and the mole fractions of the binary mixtures were calculated based on the weighed masses of NaDES and the second component.

The experimental data obtained for the binary mixtures of NaDES and water are presented in Table 2. The experimental density and dynamic viscosity values for pure water were compared with reference data available in the NIST database (Isobaric Properties for Water). The deviations obtained were 0.02% for density and 0.11% for dynamic viscosity, confirming the reliability and accuracy of the experimental measurements.

**Table 2:** Experimental Values of Density (ρ), Dynamic Viscosities (η), and Conductivities (κ) of Binary Mixtures of NaDES + Water [Water (*x*_1_)] at Various Temperatures^a^

*T* (K)					
*x*_1_	303.15	313.15	323.15	333.15	343.15
ρ (g·cm^–3^)					
0	1.4175	1.4095	1.4018	1.3937	1.3859
0.0956	1.4079	1.4004	1.3926	1.3849	1.3771
0.2030	1.3992	1.3914	1.3837	1.3760	1.3683
0.3002	1.3924	1.3845	1.3768	1.3692	1.3615
0.4005	1.3818	1.3741	1.3665	1.3588	1.3511
0.5002	1.3690	1.3616	1.3541	1.3465	1.3386
0.6001	1.3321	1.3248	1.3172	1.3095	1.3014
0.7009	1.2980	1.2903	1.2826	1.2749	1.2671
0.7968	1.2418	1.2346	1.2270	1.2197	1.2119
0.9002	1.1480	1.1419	1.1356	1.1288	1.1217
1	0.9960	0.9921	0.9881	0.9833	0.9775
η (mPa·s)					
0	7752.600	2484.900	964.450	429.480	214.530
0.0956	2101.500	814.510	357.870	175.920	95.591
0.2030	1305.000	531.410	246.030	127.590	72.417
0.3002	906.610	384.410	184.360	98.322	57.220
0.4005	466.060	214.060	109.810	61.999	37.962
0.5002	235.430	116.446	63.582	37.835	24.091
0.6001	74.986	42.303	25.965	17.039	11.817
0.7009	18.784	11.986	8.165	5.863	4.385
0.7968	8.118	5.732	4.243	3.534	2.789
0.9002	2.677	2.071	1.653	1.362	1.122
1	0.798	0.653	0.547	0.467	0.404
κ (ms·cm^–1^)					
0	0.0009	0.0014	0.0022	0.0032	0.0045
0.0956	0.0012	0.0022	0.0049	0.0087	0.0149
0.2030	0.0040	0.0079	0.0134	0.0163	0.0293
0.3002	0.0077	0.0119	0.0219	0.0261	0.0333
0.4005	0.0120	0.0227	0.0330	0.0485	0.0691
0.5002	0.0656	0.0896	0.1356	0.1678	0.1848
0.6001	0.1752	0.2607	0.3180	0.3503	0.4233
0.7009	0.4563	0.5627	0.7093	0.8070	0.8590
0.7968	1.2067	1.3440	1.7430	2.0467	2.1633
0.9002	3.1600	3.7133	4.1833	4.3787	4.5733
1	0	0	0	0	0

a Accuracy of ±0.01 K for temperature, ±0.0005 g/cm^3^ for density, and for relative viscosity ±0.35%. (0.95 level of confidence).

Figure 2 illustrates the relative deviations between the experimental data obtained in this work and the reference values available in the NIST database for both properties as a function of temperature.

**Figure 2:**
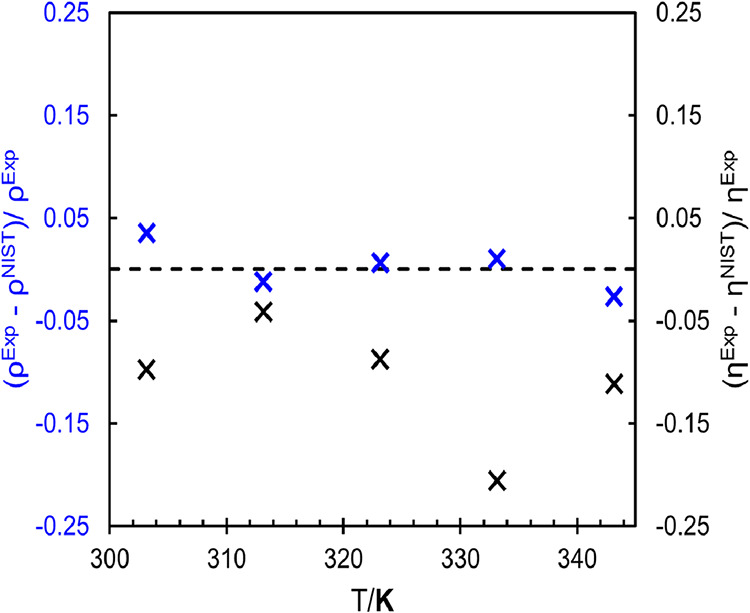
Relative deviations between the experimental data obtained in this work and the reference values from the NIST database as a function of temperature. Blue (×) symbols represent the relative deviations for density, while black (×) symbols correspond to the relative deviations for dynamic viscosity. The dotted line indicates the zero-deviation reference.

### 3.1 Density

The densities of the binary mixtures were measured under atmospheric pressure and temperatures from 303.15 to 343.15 K. The density results are presented in Figure 3.

**Figure 3:**
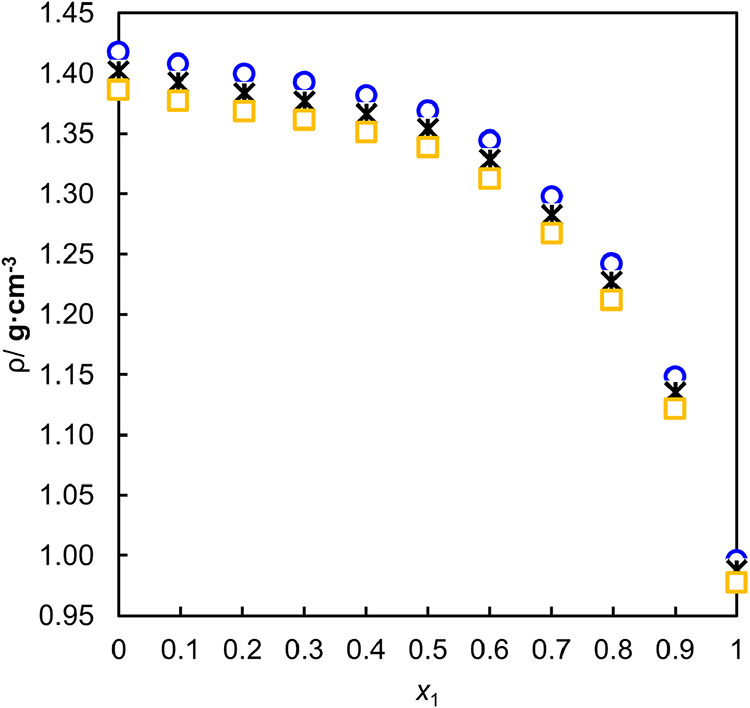
Density (ρ) as a function of the molar fraction of water (*x*_1_) for binary mixtures at different temperatures (K). The symbols correspond to experimental data obtained at □ 343.15 K, * 323.15 K, and ○ 303.15 K.

The density of each binary system decreases with increasing mole fraction of water. When *x*_1_ < 0.6, the density values decrease moderately, indicating that the intermolecular structure of the NaDES still governs the physical behavior of the system. The NaDES prepared from glycolic acid (HBD) and sorbitol (HBA) exhibits a dense hydrogen-bond network involving the hydroxyl groups of sorbitol and both the hydroxyl and carboxyl groups of glycolic acid. This extensive connectivity leads to a tightly packed liquid phase and, consequently, the mixtures initially exhibit relatively high densities.

However, as the water content increases (*x*_1_ > 0.6), a sharper decline in density is observed. This behavior indicates a gradual dismantling of the NaDES supramolecular arrangement: water molecules penetrate the acid–polyol network, competing for hydrogen-bond sites and loosening the original structure. At these higher water fractions, the system behaves essentially as a water-rich mixture, with reduced packing efficiency and weaker cohesive interactions compared than in the neat NaDES.

Additionally, for the same molar composition, density decreases with increasing temperature, a typical trend for liquids. This is attributed to the increase in molecular mobility^54^ and the expansion of free volume with temperature, leading to lower packing density in the NaDES–water mixtures.^54^

To assess whether the density variations reflect deviations from ideal mixing, we calculated the excess molar volume (*V^E^*) at all temperatures and compositions. The *V^E^* profiles help distinguish the contribution of specific interactions from effects associated with structural mismatch between the components.^55^ Water, being small and strongly polar, interacts readily with glycolic acid but does not fit as efficiently within the bulkier sorbitol framework. The disparity in molecular size, polarity, and distribution of donor and acceptor groups produces local rearrangements within the mixture,^59–61^ which is captured in both the density trends and the magnitude and sign of *V^E^*. These combined effects, competition for hydrogen-bonding sites and steric constraints, largely explain the mixing behavior observed in the glycolic acid–sorbitol–water system.

### 3.2 Excess Volume

To quantitatively describe these deviations, the excess molar volume of the binary mixtures at different temperatures was determined from the experimental density data using eq 3.^56^ In this equation, *x*_1_ represents the molar fraction of water, *M*_*1*_ and *M*_*2*_ are the molar masses of water and NaDES, respectively, and ρ, ρ_1_, and ρ_2_ denote the densities of the binary system, pure water, and pure NaDES, respectively. This calculation enables a direct correlation between the density behavior discussed above and the volumetric deviations arising from molecular interactions within the mixtures.

3 $$V^{E}=\frac{x_{1}M_{1}+\left(1-x_{1}\right)M_{2}}{\rho }-\frac{x_{1}M_{1}}{\rho_{1}}-\frac{\left(1-x_{1}\right)M_{2}}{\rho_{1}}$$

The Redlich–Kister equation was used to adjust the excess properties of the binary mixture eq 4.^57^ Δ*Q* represents the excess molar volume, *x*_1_ and *x*_2_ represent the mole fractions of water and NaDES, respectively. *A_i_* are the fitting parameters and *n* is the degree of the polynomial expansion. The adjustment parameters were obtained by the Marquardt algorithm using multiple regression analysis based on the least-squares method^58^ (Table S2 in Supporting Information).

4 $$\Delta Q=x_{1}x_{2}\sum_{i=0}^{n}A_{i}{\left(x_{1}-x_{2}\right)}^{i}$$

The standard deviation was calculated according to eq 5. With *Z* exp being the experimental values, *Z*_cal_ being the values calculated by the fitting equation and *ndat* being the number of experimental data.

5 $$\sigma ={\left[\sum_{1}^{\mathrm{ndat}}\frac{{\left(Z_{\exp}-Z_{\mathrm{cal}}\right)}^{2}}{\mathrm{ndat}}\right]}^{1/2}$$

The *V* ^*E*^ values are negative across almost the entire composition range, indicating that the mixtures experience volume contraction relative to ideal mixing. This behavior unequivocally reflects the strong specific interactions, primarily hydrogen bonding, between water molecules and the NaDES constituents.^59,60^

As can be seen in Figure 4, the excess molar volumes of binary mixtures have negative values, a behavior also observed in the work of Patyar et al.^61^ and Álvarez et al.^62^ This reduction in volume (*V* ^*E*^ < 0) results from two superimposed effects. The data used to plot the graph are presented in Table S3. First, the intramolecular incorporation of small water molecules into the relatively open hydrogen-bond network of the NaDES promotes efficient molecular packing, thereby reducing the total free volume of the mixture. Second, the strong specific hydrogen-bonding interaction between the NaDES constituents and water molecules (the chemical effect) outweighs the energy required to break the original NaDES–NaDES and water–water interactions, leading to a net increase in cohesive energy and, consequently, to volume contraction.^61^

**Figure 4:**
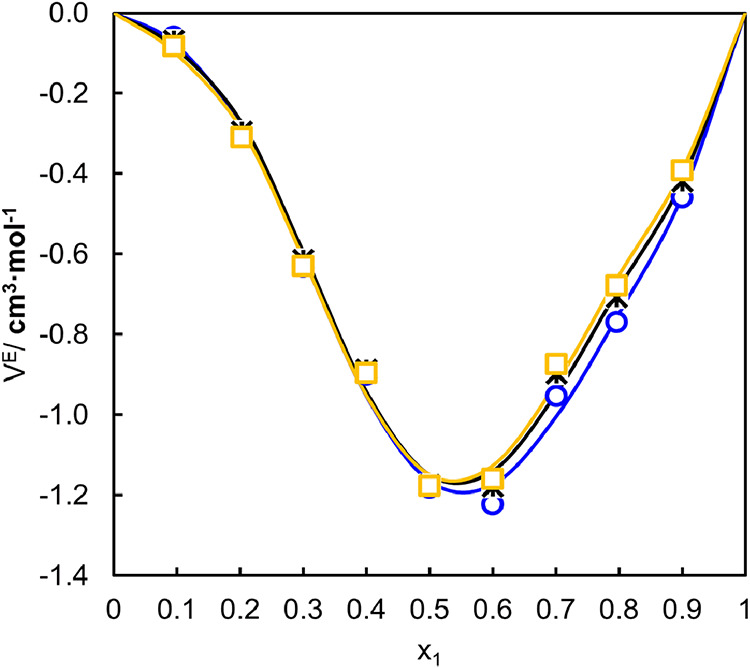
Excess molar volume (*V* ^*E*^) as a function of the molar fraction of water (*x_1_*) for the investigated binary mixtures at different temperatures (K). The symbols correspond to experimental data obtained at □ 343.15 K, * 323.15 K, and ○ 303.15 K, while the solid lines correspond to the correlation results obtained using the Redlich–Kister polynomial equation.

At lower water fractions (*x*_1_ < 0.6), the large negative *V* ^*E*^ values suggest that the NaDES structure is still largely preserved. Here, the strong formation of NaDES-H_2_O H-bonds, combined with the efficient ″fitting″ of water, enhances molecular packing and results in stronger intermolecular interactions than in the ideal state.

As the water fraction increases (*x*_1_ > 0.6), *V* ^*E*^ becomes less negative, indicating a progressive weakening of the NaDES supramolecular arrangement.^63^ As more water is incorporated into the system, these molecules begin to compete with the original HBD–HBA interactions, progressively weakening the NaDES hydrogen-bond network. This disruption reduces the cohesive forces within the liquid and creates additional free volume. The behavior is consistent with the density results shown in Figure 3, which exhibit a pronounced drop for compositions with *x*_*1*_ *> 0.6*, signaling the onset of a structural rearrangement within the mixture.

Temperature has a more modest, yet still clear, influence on the excess molar volume. As the system is heated, *V^E^* becomes slightly less negative, a consequence of enhanced molecular mobility and thermal expansion that diminishes the efficiency of molecular packing.

In summary, the negative excess molar volumes observed across the entire compositional range highlight the predominance of strong hydrogen bonding interactions in glycolic acid-sorbitol-water mixtures. The gradual shift to less negative *V^E^* values at high water concentrations signals the structural transition from a NaDES-dominated network to an aqueous environment, illustrating the crucial interplay between specific interactions (hydrogen bonds) and steric effects that govern the behavior of this binary system.

These trends are well captured by the Redlich–Kister fitting. As observed in Figure 4, the calculated curves closely follow the experimental points over the entire composition range, confirming the excellent quality of the correlation. The fitted parameters reproduce the temperature-dependent deviations from ideality and support the suitability of the model for describing the mixing behavior of these binary systems.

### 3.3 Viscosity

Viscosity measurements of binary mixtures were carried out under atmospheric pressure conditions and at temperatures ranging from 303.15 to 343.15 K. The results of these measurements are illustrated in Figure 5.

**Figure 5:**
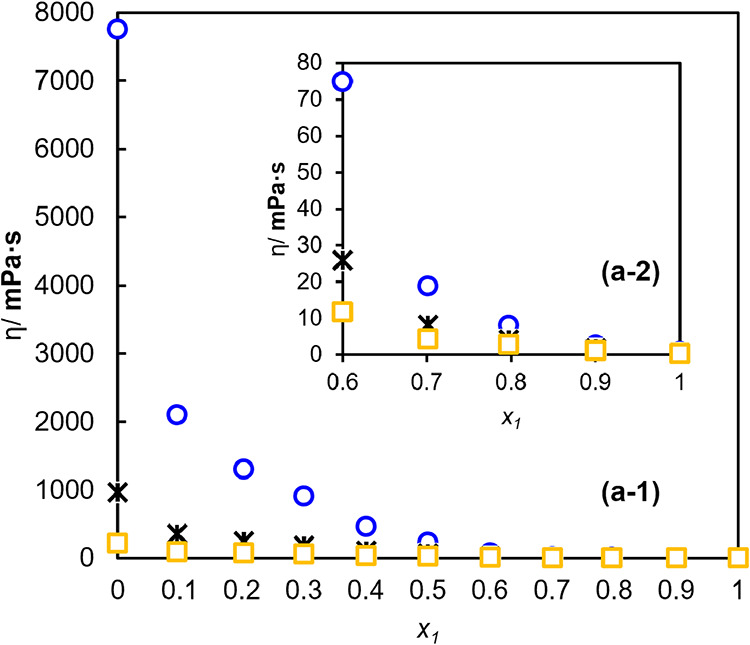
Viscosity (η) as a function of the molar fraction of water (*x*_1_) for binary mixtures at different temperatures (K). The symbols correspond to experimental data obtained at □ 343.15 K, * 323.15 K, and ○ 303.15 K.

It is possible to notice that the viscosity values of binary mixtures decrease as the mole fraction of water increases. This same pattern of behavior is observed when the temperature increases. This relationship was observed in aqueous solutions of different ILs, revealing that the viscosity decreases as the mole fraction of water increases.^64–66^ This occurs because the addition of water to pure NaDES causes the formation of hydrogen bonds between NaDES and water.^65^

### 3.4 Viscosity Deviation

Using experimental viscosity data, the viscosity deviation for binary mixtures is determined, at various temperatures, by applying eq 6. η is the viscosity of binary mixtures, *x*_1_ is the molar fraction of water, *x*_2_ is the molar fraction of NaDES, η_1_ is the viscosity of water, and η_2_ is the viscosity of NaDES.

6 $$\Delta \eta =\eta -\left(x_{1}\eta_{1}+x_{2}\eta_{2}\right)$$

As shown in Figure 6, the viscosity deviation (Δη) values are negative over the entire composition range at all investigated temperatures. The negative deviations indicate that the interactions between unlike molecules are weaker than those present in the pure components.^67^ This weakening of the intermolecular network results in a mixture that flows more easily than predicted by ideal mixing, leading to negative Δη values.^22,23^ Regarding the effect of temperature, the magnitude of the viscosity deviation becomes less negative as temperature increases, which is explained by the enhanced molecular mobility at higher temperatures, reducing the structural organization of the mixture and diminishes the real and ideal behavior.^64,65^ The data used to plot the graph are presented in Table S4.

**Figure 6:**
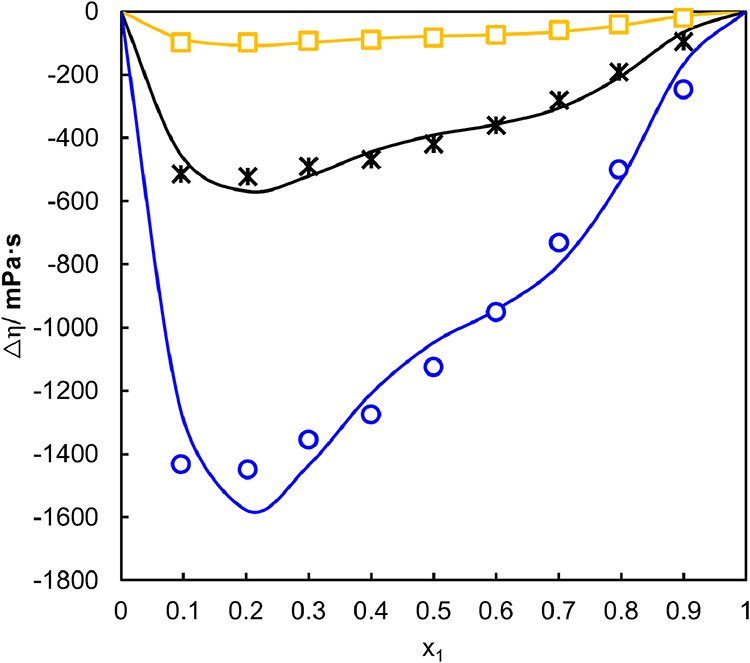
Viscosity deviation (Δη) as a function of the water mole fraction (*x*_1_) for binary mixtures at different temperatures (K). The symbols correspond to experimental data obtained at □ 343.15 K, * 323.15 K, and ○ 313.15 K, while the solid lines correspond to the correlation results obtained using the Redlich–Kister polynomial equation.

The addition of water disrupts the extensive hydrogen-bond network originally present in the NaDES formed by glycolic acid and sorbitol. As water molecules penetrate this structure, they compete for hydrogen-bonding sites, weakening the original intermolecular interactions and increasing molecular mobility within the mixture. This structural rearrangement leads to a decrease in resistance to flow, which is reflected in the negative viscosity deviations observed.

The magnitude of Δη becomes more pronounced in the intermediate composition region, indicating the maximum structural perturbation of the NaDES network. The solid lines in Figure 6 represent the correlation obtained using the Redlich–Kister polynomial equation, which reproduces the experimental data satisfactorily across the entire composition range.

The adjustment parameters used in the Redlich–Kister Equation to obtain the viscosity deviation are presented in Table S5 in the Supporting Information.

### 3.5 Conductivity

Figure 7 presents the data obtained for the conductivity of investigated binary mixtures. The analysis of conductivity values in relation to water content was adjusted by the Castel–Aymis empirical equation (eq 7). This model has been previously used to describe conductivity-composition relationships in liquid mixtures presenting a maximum in conductivity as a function of composition.^68^

**Figure 7:**
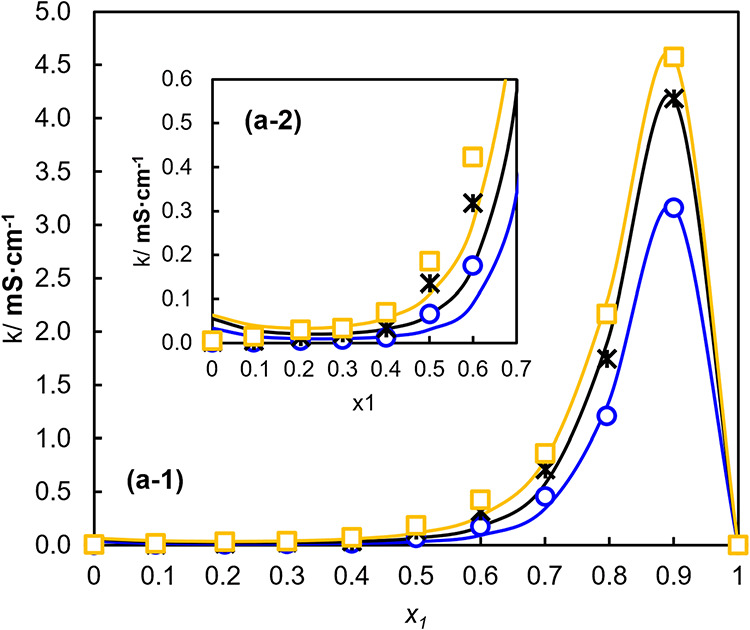
Conductivity (k) as a function of the molar fraction of water (x_1_) for binary mixtures at different temperatures (K). The symbols correspond to experimental data obtained at □ 343.15 K, * 323.15 K, and ○ 303.15 K, while the solid lines represent the correlations obtained using the Castel–Aymis equation (eq 7).

In this equation, κ represents the electrical conductivity of the mixture, κ_max_ is the maximum conductivity value of the mixture for the fraction molar of *x*_max_, and *x*_1_ is the mole fraction of water. The parameters “*a*” and “*b*” are adjustable coefficients obtained by nonlinear regression and describe the shape and curvature of the conductivity-composition profile around the maximum.

7 $$k=k_{\max}{\left(\frac{1-x_{1}}{1-x_{\max}}\right)}^{a}\exp\left[b{\left(x_{\max}-x_{1}\right)}^{2}-\frac{a}{1-x_{\max}}\left(x_{\max}-x_{1}\right)\right]$$

An examination of Figure 7 reveals that the curve can be clearly divided into two regions, separated by its maximum. Both the rising and falling portions of the curve are strongly influenced by the amount of water in the mixture. As water content increases, the electrical conductivity also rises sharply. This behavior is consistent with the enhanced mobility of charge carriers caused by the decrease in viscosity at higher water concentrations.^69^ The same trend of increase in conductivity is also observed for temperature variation.

Table 3 shows the adjustment parameters used in the Castel–Aymis equation to obtain conductivity. The standard deviation was calculated according to eq 5. A1 and A2 correspond to parameters *a* and *b* of eq 7.

**Table 3:** Fitting Parameters of Equation 7 for Conductivity Results^a^

	*T* (K)				
parameters	303.15	313.15	323.15	333.15	343.15
*x*_max_	0.900	0.900	0.900	0.900	0.900
*K*_max_	3.160	3.713	4.183	4.379	4.573
*A*1	3.494	3.594	3.100	2.741	2.682
*A*2	23.338	24.400	20.344	17.252	16.941
∑	0.009	0.026	0.021	0.011	0.016

a Accuracy of ±0.01 K for temperature.

## 4 Spectroscopic Analysis of Interactions

### 4.1 NaDES FTIR

Figure 8 presents the infrared spectrum of pure NaDES and binary mixtures of NaDES + water at different compositions. Analysis of the infrared spectrum of pure NaDES, composed of glycolic acid and sorbitol, reveals characteristic vibrational bands associated with the extensive hydrogen-bond network present in the system. In the region between 3200 cm^–1^ and 3600 cm^–1^, a broad band is observed, corresponding to the O–H stretching vibrations of hydroxyl groups involved in hydrogen bonding. This band is centered at approximately **∼3330 cm** ^**–1**^ in the pure NaDES. The bands between 2850 cm^–1^ and 3000 cm^–1^ correspond to the stretching vibrations of C–H bonds, characteristic of the carboxyl group. In a frequency range between 1650 cm^–1^ and 1800 cm^–1^, an intense and clear band stands out that can be attributed to the stretching of the carbonyl bond (C=O) of glycolic acid is observed, centered near **∼1700 cm** ^**–1**^. Furthermore, the bands between 1000 cm^–1^ and 1400 cm^–1^ are associated with C–O stretching vibrations present in the system.

**Figure 8:**
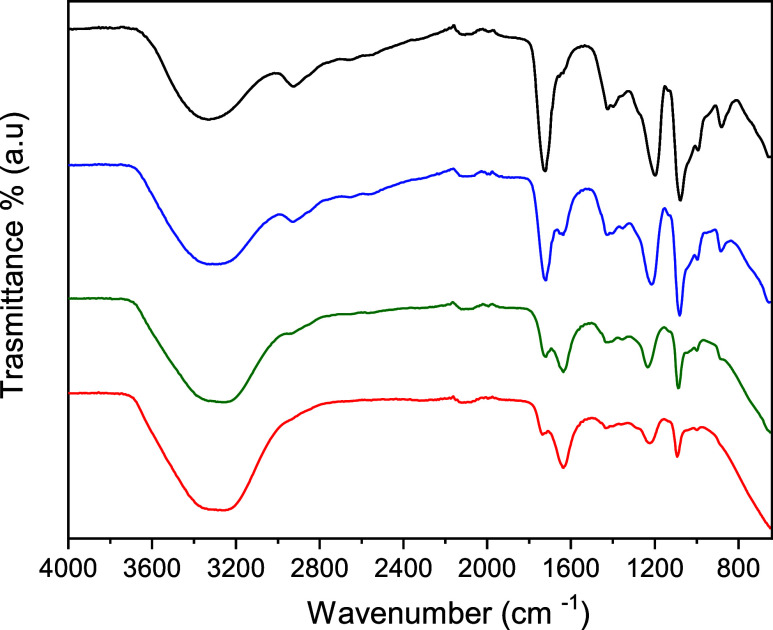
Infrared spectra of pure NaDES (black) and mixtures of [water (*X*_1_) + NaDES (1 – *X*_1_)]. Blue: *X*_1_ = 0.7009; Green: *X*_1_ = 0.9002; Red: *X*_1_ = 0.9493.

The introduction of water into the NaDES system results in noticeable modifications in the infrared spectrum. In particular, the broad O–H stretching band becomes wider and slightly shifts toward higher wavenumbers, from approximately **∼3330 cm** ^**–1**^ **in the pure NaDES to around ∼3400 cm** ^**–1**^ at higher water contents. This shift indicates a progressive modification of the hydrogen-bond network, suggesting that water molecules interact with the original hydrogen-bond donor and acceptor groups of the NaDES components and partially disrupt the supramolecular structure formed by glycolic acid and sorbitol.

Furthermore, in the region between 2850 and 3000 cm^–1^, the band attributed to C–H stretching vibrations shows a gradual decrease in intensity as the water fraction increases,^16^ reflecting changes in the local molecular environment of the NaDES components. Similarly, the **C**=**O stretching band near ∼1700 cm** ^**–1**^ progressively decreases in intensity with increasing water content, indicating modifications in the hydrogen-bond interactions involving the carbonyl group.

The bands associated with **C–O stretching vibrations in the 1000–1400 cm** ^**–1**^ **region** also decrease in intensity as the NaDES fraction decreases in the mixture, indicating a progressive disruption of the original NaDES supramolecular arrangement.

At high water content (*X*_1_ = 0.9493), a band characteristic of water becomes more pronounced in the **∼1640 cm** ^**–1**^ **region**, corresponding to the H–O–H bending vibration. This behavior indicates that, at high water fractions, the spectral profile becomes increasingly dominated by vibrational modes associated with water, reflecting the gradual transition from a NaDES-dominated hydrogen-bond network to a water-rich environment.

## 5 MD Simulations

### 5.1 Validation of MD Simulations

To validate the parameters used to describe the sorbitol, glycolic acid, and water molecules in the MD simulations, the density values obtained from these simulations were correlated with the density values from experimental systems at 303.15, 323.15, and 343.15 K, as shown in Table 4. Analyzing the increase in temperature, a decrease in the density values in all systems analyzed. Furthermore, analyzing the density values obtained using both approaches (experimental and MD simulations), MD simulations registered density values close to experimental values in all systems analyzed, with low error. Therefore, the parameters used for the SOR, GLY, and WAT molecules can be used to describe the interactions between these components.

**Table 4:** Density Values Obtained Using Experimental Systems and MD Simulations at Temperatures of 303.15, 323.15, and 343.15 K

systems	temperature/K	experimental/g cm^–3^	simulated/g cm^–3^	error/%
NaDESs	303.15	1.4175	1.3851	2.29
	323.15	1.4018	1.3630	2.77
	343.15	1.3859	1.3377	3.48
NaDESs-Water	303.15	1.3690	1.3124	4.13
	323.15	1.3541	1.2924	4.55
	343.15	1.3386	1.2701	5.11
Water	303.15	0.9960	0.9962	0.02
	323.15	0.9881	0.9843	0.38
	343.15	0.9775	0.9702	0.75

### 5.2 Hydrogen Bonds and IPE Analysis

To analyze the effects of temperature increases on NaDESs, NaDESs-Water, and Water systems, the hydrogen bond analysis (Table 5) and IPE analysis (Table 6) were performed between the components present in the systems. Regarding the hydrogen bond analysis, the highest number of hydrogen bonds was observed between WAT molecules in the Water system, regardless of the temperature employed. This highest number of hydrogen bonds may be explained by the high amount of WAT in this system, as well as the small size of WAT molecules, with the absence of relevant steric effects. The second-highest number of hydrogen bonds was observed between the SOR and GLY molecules in the NaDESs system, regardless of the temperature analyzed. This may be explained by the fact that SOR and GLY have 12 and five hydrogen bond sites (Figure S3 in the Supporting Information), respectively. Analyzing the increase in temperature, it was observed that the number of hydrogen bonds in all interactions (except the WAT-WAT interaction in the NaDESs-Water system, which remains almost constant) decreased, indicating the separation of molecules from each other.

**Table 5:** Hydrogen Bonds with Standard Deviation between the Components in the NaDES, NaDESs-Water, and Water Systems at Temperatures of 303.15, 323.15, and 343.15 K

hydrogen bonds						
	NaDESs	NaDESs-water				water
*T* (K)	SOR-GLY	SOR-GLY	SOR-WAT	GLY-WAT	WAT-WAT	WAT-WAT
303.15	9073.22 (±82.92)	4913.48 (±65.48)	5090.52 (±59.33)	7873.39 (±77.23)	3916.21 (±51.06)	59311.12 (±82.65)
323.15	8957.61 (±72.33)	4710.03 (±58.84)	4974.67 (±66.07)	7628.98 (±124.79)	3896.93 (±72.30)	57805.48 (±88.23)
343.15	8621.25 (±75.44)	4510.59 (±57.98)	4885.63 (±75.61)	7255.50 (±220.77)	3924.46 (±121.83)	56253.81 (±95.31)

**Table 6:** IPE between the Components in the NaDES, NaDESs-Water, and Water Systems at Temperatures of 303.15, 323.15, and 343.15 K

	IPE/kJ mol^–1^					
	NaDESs	NaDESs-Water				Water
*T* (K)	SOR-GLY	SOR-GLY	SOR-WAT	GLY-WAT	WAT-WAT	WAT-WAT
303.15	–428,255	–246,631	–134,852	–260,779	–117,086	–1,536,200
323.15	–417,907	–235,896	–131,921	–248,460	–116,620	–1,458,730
343.15	–394,408	–223,280	–130,257	–229,841	–117,708	–1,496,980

Analyzing the IPE results in Table 6, the strongest interaction was observed between the WAT molecules in the Water system, regardless of the temperature employed. The highest hydrogen bonds observed previously (Table 5) between these molecules play an essential role in the IPE result, because this intermolecular force contributes around – 1 to – 40 kJ mol^–1^.^70^ The second strongest interaction was between the SOR and GLY components, regardless of the temperature employed. Analyzing the increase in temperature, all IPE values increased (except the WAT-WAT interaction in the NaDESs-Water and Water systems) was observed, regardless of the employed temperature. Therefore, the IPE results showed that increasing in temperature reduced affinity between components, except for WAT-WAT interactions in the NaDESs-Water and Water systems.

## 6 Conclusion

The thermophysical properties of binary mixtures of glycolic acid–sorbitol (3:1) NaDES and water were systematically investigated over a temperature range of 303.15 to 343.15 K across the full composition range. Density and viscosity decreased continuously with increasing temperature and water content, with viscosity dropping from 7752.6 mPa·s for pure NaDES at 303.15 K to 0.404 mPa·s for pure water at 343.15 K. Excess molar volume values were negative throughout the entire composition range, with the most negative values at *x*_1_ < 0.6, reflecting strong hydrogen-bonding interactions and volume contraction relative to ideal mixing. Above *x*_1_ = 0.6, *VE* became progressively less negative, consistent with the gradual disruption of the NaDES supramolecular network. Both *V^E^* and viscosity deviation were satisfactorily correlated using the Redlich–Kister equation.

Conductivity exhibited a nonmonotonic dependence on water content, reaching a maximum of approximately 4.57 mS·cm^–1^ near *x*_1_ ≈ 0.9 at 343.15 K, a trend well described by the Castel-Aymis equation. FTIR spectroscopy and molecular dynamics simulations consistently indicated a progressive weakening of the hydrogen-bond network with increasing water content and temperature. The number of SOR–GLY hydrogen bonds decreased from 9073 at 303.15 K to 8621 at 343.15 K in the pure NaDES system, accompanied by a reduction in the SOR–GLY interaction potential energy from −428.255 to −394.408 kJ·mol^–1^ over the same temperature range. These results provide a coherent molecular-level rationale for all observed macroscopic property trends in the glycolic acid–sorbitol–water system.

## Supplementary Material



## Data Availability

The data supporting this study are available within the manuscript. Additional illustrative figures and tables are provided in the Supporting Information.

## Notes

^1^H and ^13^C NMR spectra of NaDES (glycolic acid–sorbitol systems), hydrogen-bonding sites, molecular dynamics simulation details, and experimental and correlated data for excess molar volume and viscosity deviations (PDF)

## References

[1] Hansen B. B., Spittle S., Chen B., Poe D., Zhang Y., Klein J. M., Horton A., Adhikari L., Zelovich T., Doherty B. W., Gurkan B., Maginn E. J., Ragauskas A., Dadmun M., Zawodzinski T. A., Baker G. A., Tuckerman M. E., Savinell R. F., Sangoro J. R. (2021). Deep Eutectic Solvents: A Review
of Fundamentals and Applications. Chem. Rev..

[2] El
Achkar T., Greige-Gerges H., Fourmentin S. (2021). Basics and
Properties of Deep Eutectic Solvents: A Review. Environ. Chem. Lett..

[3] Lapeña D., Errazquin D., Lomba L., Lafuente C., Giner B. (2021). Ecotoxicity
and Biodegradability of Pure and Aqueous Mixtures of Deep Eutectic
Solvents: Glyceline, Ethaline, and Reline. Environ.
Sci. Pollut. Res..

[4] Ijardar S. P., Singh V., Gardas R. L. (2022). Revisiting the Physicochemical
Properties
and Applications of Deep Eutectic Solvents. Molecules.

[5] Inayat S., Ahmad S. R., Awan S. J., Nawshad M., Ali Q. (2023). In Vivo and
In Vitro Toxicity Profile of Tetrabutylammonium Bromide and Alcohol-Based
Deep Eutectic Solvents. Sci. Rep..

[6] Liu Y., Friesen J. B., McAlpine J. B., Lankin D. C., Chen S.-N., Pauli G. F. (2018). Natural Deep Eutectic Solvents: Properties, Applications,
and Perspectives. J. Nat. Prod..

[7] Vanda H., Dai Y., Wilson E. G., Verpoorte R., Choi Y. H. (2018). Green Solvents from
Ionic Liquids and Deep Eutectic Solvents to Natural Deep Eutectic
Solvents. C. R. Chim..

[8] Santana A. P. R., Mora-Vargas J. A., Guimarães T. G. S., Amaral C. D. B., Oliveira A., Gonzalez M. H. (2019). Sustainable Synthesis
of Natural Deep Eutectic Solvents (NADES) by Different Methods. J. Mol. Liq..

[9] Li Z.-L., Zhong F.-Y., Huang J.-Y., Peng H.-L., Huang K. (2020). Sugar-Based
Natural Deep Eutectic Solvents as Potential Absorbents for NH_3_ Capture at Elevated Temperatures and Reduced Pressures. J. Mol. Liq..

[10] Bajkacz S., Adamek J. (2018). Development of a Method Based on
Natural Deep Eutectic
Solvents for Extraction of Flavonoids from Food Samples. Food Anal. Methods.

[11] Yang Z. (2019). Natural Deep
Eutectic Solvents and Their Applications in Biotechnology. Adv. Biochem. Eng. Biotechnol..

[12] Pavoković D., Košpić K., Panić M., Radojčić
Redovniković I., Cvjetko Bubalo M. (2020). Natural Deep Eutectic Solvents Are
Viable Solvents for Plant Cell Culture-Assisted Stereoselective Biocatalysis. Process Biochem.

[13] Zainal-Abidin M. H., Hayyan M., Ngoh G. C., Wong W. F., Looi C. Y. (2019). Emerging
Frontiers of Deep Eutectic Solvents in Drug Discovery and Drug Delivery
Systems. J. Controlled Release.

[14] Zhang M., Zhang X., Liu Y., Wu K., Zhu Y., Lu H., Liang B. (2021). Insights into the Relationships
between Physicochemical
Properties, Solvent Performance, and Applications of Deep Eutectic
Solvents. Environ. Sci. Pollut. Res..

[15] Omar K. A., Sadeghi R. (2022). Physicochemical Properties
of Deep Eutectic Solvents:
A Review. J. Mol. Liq..

[16] Wang X., Liu B., Yang H., Tian J. (2022). Properties of Binary Mixtures of
a Novel Natural Deep Eutectic Solvent (Glycolic Acid + Xylitol) and
Water at Several Temperatures. Fluid Phase Equilib..

[17] Kuddushi M., Nangala G. S., Rajput S., Ijardar S. P., Malek N. I. (2019). Understanding
the Peculiar Effect of Water on the Physicochemical Properties of
Choline Chloride Based Deep Eutectic Solvents Theoretically and Experimentally. J. Mol. Liq..

[18] Nowosielski B., Jamrógiewicz M., Łuczak J., Warmińska D. (2022). Novel Binary
Mixtures of Alkanolamine Based Deep Eutectic Solvents with Water—Thermodynamic
Calculation and Correlation of Crucial Physicochemical Properties. Molecules.

[19] Leron R. B., Soriano A. N., Li M.-H. (2012). Densities and Refractive
Indices
of the Deep Eutectic Solvents (Choline Chloride + Ethylene Glycol
or Glycerol) and Their Aqueous Mixtures at the Temperature Ranging
from 298.15 to 333.15 K. J. Taiwan Inst. Chem.
Eng..

[20] Gajardo-Parra N. F., Lubben M. J., Winnert J. M., Leiva Á., Brennecke J. F., Canales R. I. (2019). Physicochemical Properties of Choline Chloride-Based
Deep Eutectic Solvents and Excess Properties of Their Pseudo-Binary
Mixtures with 1-Butanol. J. Chem. Thermodyn..

[21] Bakhtyari A., Haghbakhsh R., Duarte A. R. C., Raeissi S. (2020). A Simple Model for
the Viscosities of Deep Eutectic Solvents. Fluid
Phase Equilib..

[22] Gajardo-Parra N. F., Cotroneo-Figueroa V.
P., Aravena P., Vesovic V., Canales R. I. (2020). Viscosity
of Choline Chloride-Based Deep Eutectic Solvents: Experiments and
Modeling. J. Chem. Eng. Data.

[23] Ghaedi H., Akbari S., Zhou H., Wang W., Zhao M. (2022). Excess Properties
of and Simultaneous Effects of Important Parameters on CO_2_ Solubility in Binary Mixture of Water-Phosphonium Based-Deep Eutectic
Solvents: Response Surface Methodology (RSM) and Taguchi Method. Energy Fuels.

[24] Myrdek T., Popescu C., Kunz W. (2021). Physical-Chemical
Properties of Newly
Synthesized Tetraalkylammonium Alkyl Ether Carboxylate Ionic Liquids. J. Mol. Liq..

[25] Boublia A., Lemaoui T., Abu Hatab F., Darwish A. S., Banat F., Benguerba Y., AlNashef I. M. (2022). Molecular-Based Artificial Neural
Network for Predicting the Electrical Conductivity of Deep Eutectic
Solvents. J. Mol. Liq..

[26] Kovács A., Neyts E. C., Cornet I., Wijnants M., Billen P. (2020). Modeling the
Physicochemical Properties of Natural Deep Eutectic Solvents. ChemSusChem.

[27] Sun H., Li Y., Wu X., Li G. (2013). Theoretical Study on the Structures
and Properties of Mixtures of Urea and Choline Chloride. J. Mol. Model..

[28] Mainberger S., Kindlein M., Bezold F., Elts E., Minceva M., Briesen H. (2017). Deep Eutectic Solvent Formation:
A Structural View
Using Molecular Dynamics Simulations with Classical Force Fields. Mol. Phys..

[29] Perkins S. L., Painter P., Colina C. M. (2014). Experimental and
Computational Studies
of Choline Chloride-Based Deep Eutectic Solvents. J. Chem. Eng. Data.

[30] Kaur S., Sharma S., Kashyap H. K. (2017). Bulk and Interfacial Structures of
Reline Deep Eutectic Solvent: A Molecular Dynamics Study. J. Chem. Phys..

[31] Bittner J. P., Smirnova I., Jakobtorweihen S. (2024). Investigating Biomolecules in Deep
Eutectic Solvents with Molecular Dynamics Simulations: Current State,
Challenges and Future Perspectives. Molecules.

[32] Lane J. N., Klimkowski V. J., Hopkins T. A. (2025). Molecular Dynamics Investigation
of Deep Eutectic Solvent Structure and Properties Based on Hydrogen
Bond Acceptor Variation. J. Mol. Liq..

[33] Shah D., Mjalli F. S. (2014). Effect of Water
on the Thermo-Physical Properties of
Reline: An Experimental and Molecular Simulation Based Approach. Phys. Chem. Chem. Phys..

[34] Kumari P., Shobhna, Kaur S., Kashyap H. K. (2018). Influence of Hydration on the Structure of Reline Deep
Eutectic Solvent: A Molecular Dynamics Study. ACS Omega.

[35] Baz J., Held C., Pleiss J., Hansen N. (2019). Thermophysical Properties
of Glyceline–Water Mixtures Investigated by Molecular Modelling. Phys. Chem. Chem. Phys..

[36] Ahmadi R., Hemmateenejad B., Safavi A., Shojaeifard Z., Shahsavar A., Mohajeri A., Dokoohaki M. H., Zolghadr A. R. (2018). Deep Eutectic–Water Binary Solvent Associations
Investigated by Vibrational Spectroscopy and Chemometrics. Phys. Chem. Chem. Phys..

[37] Zhekenov T., Toksanbayev N., Kazakbayeva Z., Shah D., Mjalli F. S. (2017). Deep Eutectic
Solvents and Effect of Water on Their Intermolecular Interactions. Fluid Phase Equilib..

[38] Abbott A. P., Boothby D., Capper G., Davies D. L., Rasheed R. K. (2004). Deep Eutectic
Solvents Formed between Choline Chloride and Carboxylic Acids: Versatile
Alternatives to Ionic Liquids. J. Am. Chem.
Soc..

[39] Liu B., Tian J. (2021). Investigation of Glycolic
Acid Natural Deep Eutectic Solvents with
Strong Proton Donors for Ammonia Capture and Separation. Ind. Eng. Chem. Res..

[40] Becke A. D. (1993). Density-Functional
Thermochemistry. III. The Role of Exact Exchange. J. Chem. Phys..

[41] Lee C., Yang W., Parr R. G. (1988). Development of the Colle-Salvetti
Correlation-Energy Formula into a Functional of the Electron Density. Phys. Rev. B.

[42] Vosko S. H., Wilk L., Nusair M. (1980). Accurate Spin-Dependent Electron
Liquid Correlation Energies for Local Spin Density Calculations: A
Critical Analysis. Can. J. Phys..

[43] Hehre W. J., Ditchfield R., Pople J. A. (1972). Self-Consistent Molecular Orbital
Methods. XII. Further Extensions of Gaussian-Type Basis Sets for Use
in Molecular Orbital Studies of Organic Molecules. J. Chem. Phys..

[44] Frisch, M. J. ; Trucks, G. W. ; Schlegel, H. B. ; Scuseria, G. E. ; Robb, M. A. ; Cheeseman, J. R. ; Scalmani, G. ; Barone, V. ; Petersson, G. A. ; Nakatsuji, H. Gaussian 09, Revision A.02; Gaussian, Inc.: Wallingford, CT, 2016.

[45] Abraham M. J., Murtola T., Schulz R., Páll S., Smith J. C., Hess B., Lindahl E. (2015). GROMACS: High
Performance
Molecular Simulations through Multi-Level Parallelism from Laptops
to Supercomputers. SoftwareX.

[46] Jorgensen W. L., Maxwell D. S., Tirado-Rives J. (1996). Development
and Testing of the OPLS
All-Atom Force Field on Conformational Energetics and Properties of
Organic Liquids. J. Am. Chem. Soc..

[47] Mark P., Nilsson L. (2001). Structure and Dynamics
of the TIP3P, SPC, and SPC/E
Water Models at 298 K. J. Phys. Chem. A.

[48] Haug E. J., Arora J. S., Matsui K. (1976). A Steepest-Descent
Method for Optimization
of Mechanical Systems. J. Optim. Theory Appl..

[49] Yuan G., Li T., Hu W. (2019). A Conjugate
Gradient Algorithm and Its Application
in Large-Scale Optimization Problems and Image Restoration. J. Inequal. Appl..

[50] Bussi G., Donadio D., Parrinello M. (2007). Canonical
Sampling through Velocity
Rescaling. J. Chem. Phys..

[51] Bernetti M., Bussi G. (2020). Pressure Control Using
Stochastic Cell Rescaling. J. Chem. Phys..

[52] van
Gunsteren W. F., Berendsen H. J. C. (1988). A Leap-Frog Algorithm for Stochastic
Dynamics. Mol. Simul..

[53] Amorim-Carmo B., Daniele-Silva A., Parente A. M. S., Furtado A. A., Carvalho E., Oliveira J. W. F., Elizabeth C. G., Silva M. S., Silva S. R. B., Arnóbio A. A., Monteiro N. K., Fernandes-Pedrosa M.
F. (2019). Potent
and Broad-Spectrum Antimicrobial Activity of Analogs from the Scorpion
Peptide Stigmurin. Int. J. Mol. Sci..

[54] Bergua F., Nuez M., Muñoz-Embid J., Lafuente C., Artal M. (2018). Volumetric
and Acoustic Behaviour of Myo-Inositol in Aqueous Natural Deep Eutectic
Solvent Solutions. J. Mol. Liq..

[55] Haghbakhsh R., Duarte A. R. C., Raeissi S. (2021). Volumetric
Investigation of Aqueous
Mixtures of the {Choline Chloride + Phenol (1:4)} Deep Eutectic Solvent. J. Chem. Thermodyn..

[56] Bahadur I., Letcher T. M., Singh S., Redhi G. G., Venkatesu P., Ramjugernath D. (2015). Excess Molar Volumes of Binary Mixtures
(an Ionic Liquid
+ Water): A Review. J. Chem. Thermodyn..

[57] Rodríguez H., Brennecke J. F. (2006). Temperature
and Composition Dependence of the Density
and Viscosity of Binary Mixtures of Water + Ionic Liquid. J. Chem. Eng. Data.

[58] Agca C., McMurray J. W. (2022). Empirical Estimation of Densities in NaCl-KCl-UCl_3_ and NaCl-KCl-YCl_3_ Molten Salts Using Redlich-Kister
Expansion. Chem. Eng. Sci..

[59] Ghaedi H., Ayoub M., Sufian S., Shariff A. M., Murshid G., Hailegiorgis S. M., Khan S. N. (2017). Density, Excess and Limiting Properties
of (Water and Deep Eutectic Solvent) Systems at Temperatures from
293.15 to 343.15 K. J. Mol. Liq..

[60] Abbott A. P., Capper G., Davies D. L., Rasheed R. K., Tambyrajah V. (2003). Novel Solvent
Properties of Choline Chloride/Urea Mixtures. Chem. Commun..

[61] Patyar P., Ali A., Malek N. I. (2021). Experimental
and Theoretical Excess Molar Properties
of Aqueous Choline Chloride Based Deep Eutectic Solvents. J. Mol. Liq..

[62] Álvarez M. S., Deive F. J., Longo M. A., Rodríguez A., Segade L., Cabeza O. (2023). Physico-Chemical Characterization
of Methanolic Mixtures of Cholinium Dihydrogen Phosphate-Based DES. J. Mol. Liq..

[63] Hammond O. S., Bowron D. T., Edler K. J. (2017). The Effect of Water
upon Deep Eutectic
Solvent Nanostructure: An Unusual Transition from Ionic Mixture to
Aqueous Solution. Angew. Chem., Int. Ed..

[64] Terdale S. S., Warke I. J. (2020). Physicochemical
Properties of Dilute Aqueous Solutions
of 1-Ethyl-3-Methylimidazolium Ethylsulfate, 1-Ethyl-3-Methylimidazolium
Methylsulfate, 1-Ethyl-3-Methylimidazolium Tosylate and 1,3-Dimethylimidazolium
Methylsulfate at Different Temperatures and at Atmospheric Pressure. J. Chem. Thermodyn..

[65] Zhang F. F., Li X. Y., Chen G., Wang T., Jin T. X., Cheng C. X., Li G. P., Zhang L. G., Zhang B., Zheng F. F. (2021). Thermophysical Properties and Water
Sorption Characteristics
of 1-Ethyl-3-Methylimidazolium Acetate Ionic Liquid and Water Binary
Systems. Int. Commun. Heat Mass Transfer.

[66] Horwitz G., Steinberg P. Y., Corti H. R. (2021). Volumetric and Viscosity Properties
of Water-in-Salt Lithium Electrolytes: A Comparison with Ionic Liquids
and Hydrated Molten Salts. J. Chem. Thermodyn..

[67] Liu Q., Ma L., Wang S., Ni Z., Fu X., Wang J., Zheng Q. (2021). Study on the Properties
of Density, Viscosity, Excess Molar Volume,
and Viscosity Deviation of [C2mim]­[NTf2], [C2mmim]­[NTf2], [C4mim]­[NTf2],
and [C4mmim]­[NTf2] with PC Binary Mixtures. J. Mol. Liq..

[68] Wei Y., Jin Y., Wu Z.-J., Yang Y., Zhang Q.-G., Kang Z.-H. (2013). Synthesis
and Physicochemical Properties of Amino Acid Ionic Liquid 1-Butyl-3-methylimidazolium
Aspartate and Binary Mixture with Methanol. J. Chem. Eng. Data.

[69] Zhang Q.-G., Sun S.-S., Pitula S., Liu Q.-S., Welz-Biermann U., Zhang J.-J. (2011). Electrical Conductivity of Solutions of Ionic Liquids
with Methanol, Ethanol, Acetonitrile, and Propylene Carbonate. J. Chem. Eng. Data.

[70] Izgorodina E. I., MacFarlane D. R. (2011). Nature of Hydrogen Bonding in Charged Hydrogen-Bonded
Complexes and Imidazolium-Based Ionic Liquids. J. Phys. Chem. B.

