# Supplementary material for: Thermophysical Properties and Molecular Dynamics Insights into Glycolic Acid–Sorbitol NaDES–Water Mixtures
Source: ACS Omega. 2026 May 2;11(18):27092–102. doi: 10.1021/acsomega.6c00721 (PMC13176986; doi:10.1021/acsomega.6c00721)
Supplement: Supplementary file 1 [file ao6c00721_si_001.pdf]

Thermophysical Properties and Molecular Dynamics Insights into Glycolic Acid–Sorbitol NaDES–Water Mixtures

Marcelo M. do Ó[1], Sádwa F. Ribeiro[1], Lucas L. Bezerra[2], Norberto de K. V. Monteiro[2], Alanderson A. A. Alves[1], Francisco H. B. Quinto[1], Rilvia S. de Santiago-Aguiar[1,*]

FIGURES

Figure S1. ^{1}H NMR spectra in DMSO-d6 (which was used as a solvent) for NaDES (Glycolic acid and sorbitol): a) simple mixture (SM) and b) DES [GA: SOR (3:1)].

Figure S1a - ^{1}H NMR spectra of the simple mixture of molecules (Glycolic acid (HBD) and sorbitol (HBA));

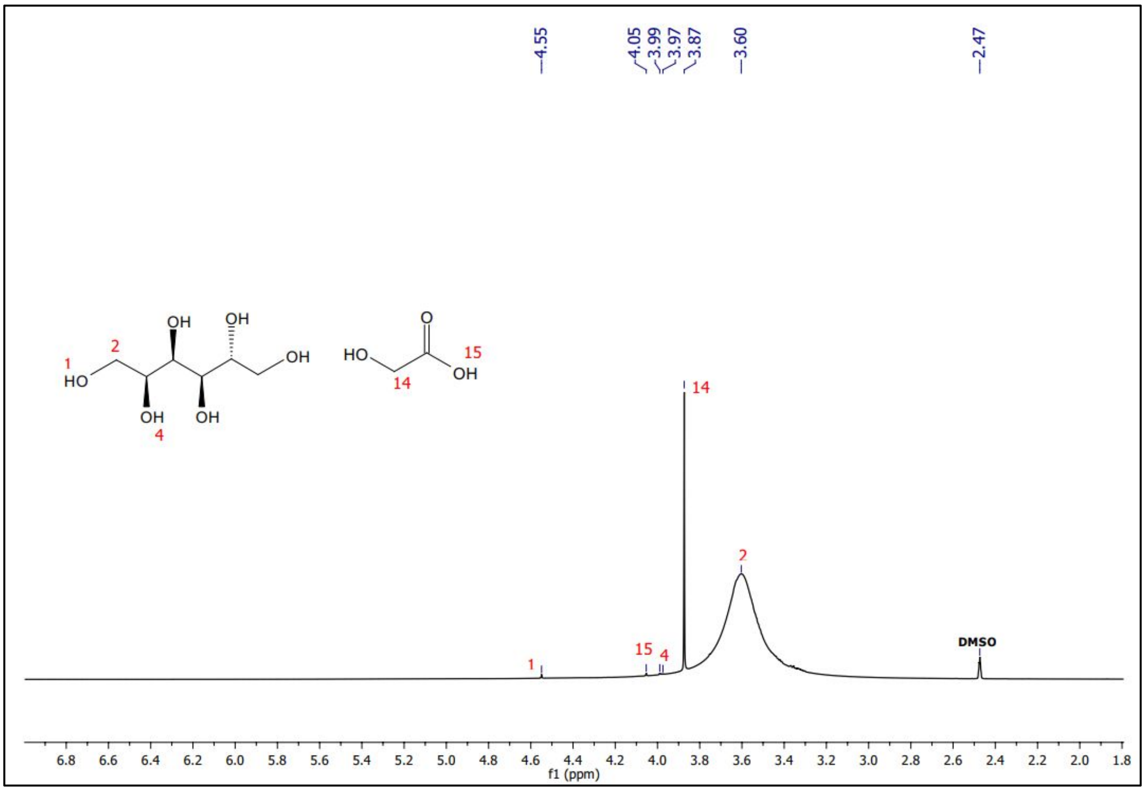

Figure S1b. ^{1}H NMR spectra of the NaDES molecules (Glycolic acid (HBD) and sorbitol (HBA));

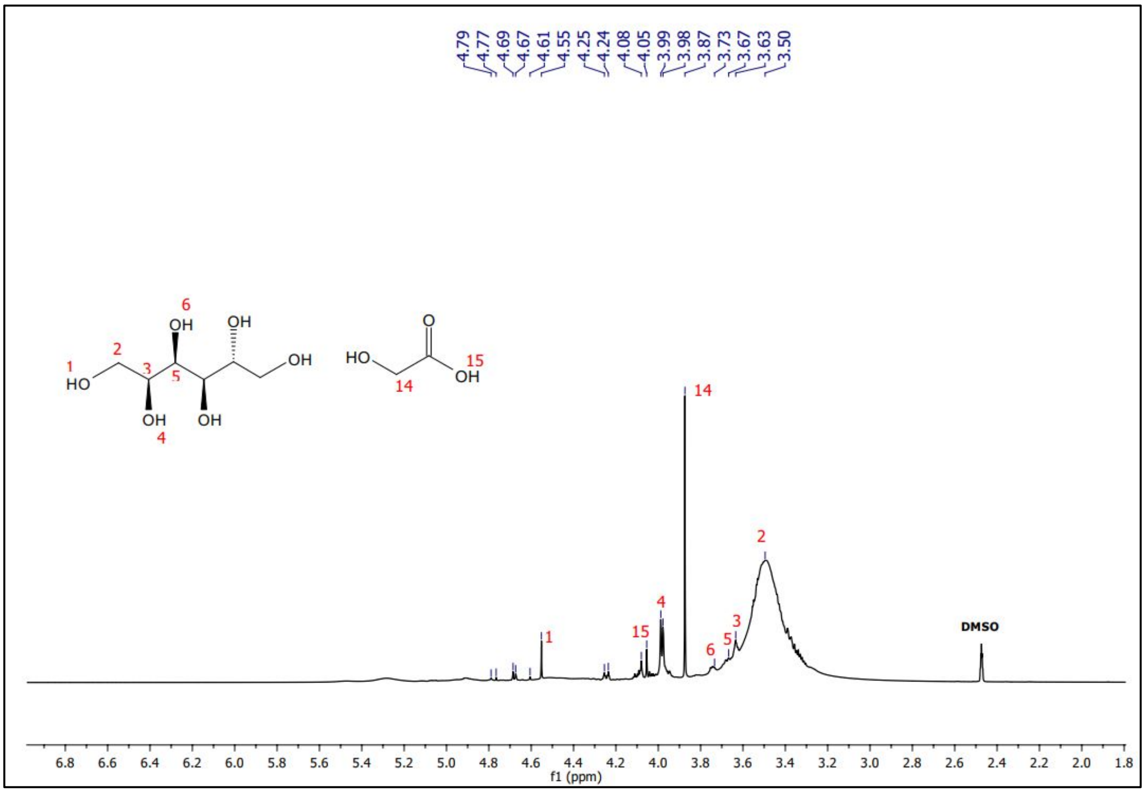

Figure S2. ^{13}C NMR spectra of the NaDES molecules (Glycolic acid (HBD) and sorbitol (HBA));

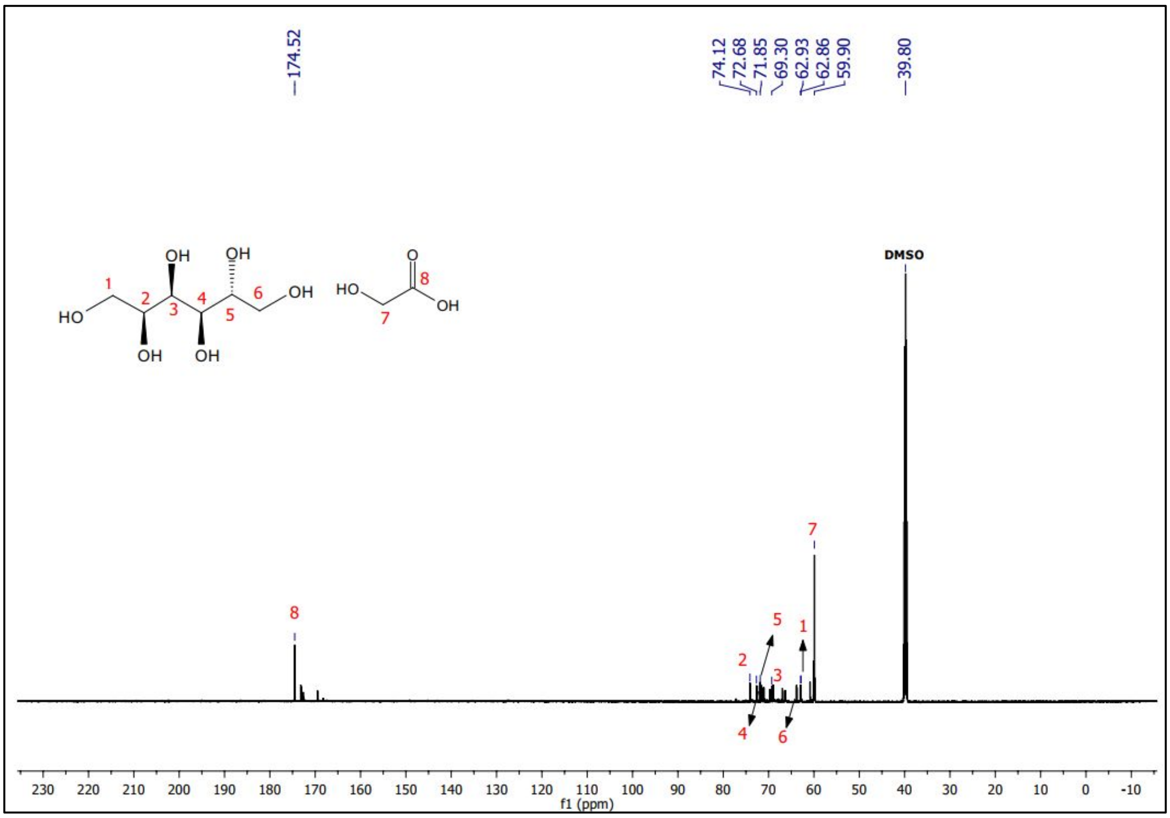

Figure S3. Hydrogen bond sites for the (a) SOR and (b) GLY molecules.

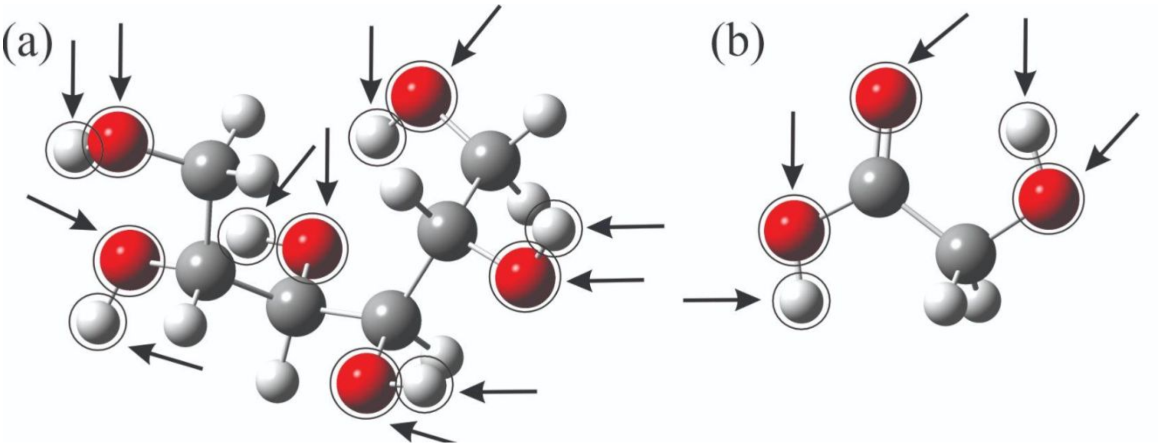

TABLES

Table S1. Number of species employed in the MD simulations based on the experimental section.

Systems	SOR	GLY	WAT
NaDESs ($X_1 = 0.0$)	2077	6231	
NaDESs-Water ($X_1 = 0.0052$)	1701	5103	6622
Water ($X_1 = 1.0$)	-	-	33110

Table S2. Adjustment parameters of Equation 2 for excess molar volume (V^E) results[a]

Parameters	T (K)				
	303.15	313.15	323.15	333.15	343.15
$\boldsymbol{A_0}$	-4.6630	-4.7825	-4.6005	-4.7371	-4.5965
$\boldsymbol{A_1}$	-4.7827	-11.2650	-8.1437	-12.0705	-8.4555
$\boldsymbol{A_2}$	23.1566	34.0766	29.3768	35.6563	29.6796
$\boldsymbol{A_3}$	-0.8589	-0.7898	-0.8334	-0.7917	-0.8329
$\boldsymbol{A_4}$	-0.2565	-0.2454	-0.2540	-0.2454	-0.2534
σ	0.014	0.014	0.015	0.015	0.016
AARD	0.062	0.083	0.059	0.079	0.051

[a]Accuracy of ± 0.01 K for temperature.

Table S3. Experimental Excess Molar Volume (V^E) for Water and Binary Mixtures of NADES + Water at Various Temperatures, Water Mole Fraction (x_1) [a]

x_i	303.15	313.15	323.15	333.15	343.15
0.00	0.00	0.00	0.00	0.00	0.00
0.10	-0.06	-0.08	-0.07	-0.08	-0.08
0.20	-0.30	-0.30	-0.29	-0.31	-0.31
0.30	-0.63	-0.62	-0.61	-0.63	-0.63
0.40	-0.90	-0.90	-0.89	-0.89	-0.90
0.50	-1.18	-1.18	-1.18	-1.18	-1.18
0.60	-1.22	-1.31	-1.18	-1.27	-1.16
0.70	-0.95	-0.93	-0.90	-0.88	-0.87
0.80	-0.77	-0.74	-0.71	-0.69	-0.68
0.90	-0.46	-0.44	-0.42	-0.40	-0.39
1.00	0.00	0.00	0.00	0.00	0.00

[a] Accuracy of ± 0.01 K for temperature, and ± 0.0005 g/cm^3 for density (0.95 level of confidence).
Combined uncertainty for excess molar volume $u(V^E)$ = 0.05× 10^{-} m^3 mol^{-1}

Table S4. Experimental Viscosity Deviation (Δη) for Water and Binary Mixtures of NADES + Water at Various Temperatures Water Mole Fraction (x_1) [a]

x_i	303.15	313.15	323.15	333.15	343.15
0.00	0.0	0.0	0.0	0.0	0.0
0.10	-4909.7	-1432.8	-514.4	-212.5	-98.5
0.20	-4873.6	-1449.1	-522.7	-214.8	-98.6
0.30	-4519.0	-1354.7	-490.7	-202.4	-93.0
0.40	-4181.6	-1275.8	-468.6	-195.6	-90.8
0.50	-3639.7	-1125.8	-418.7	-177.1	-83.3
0.60	-3026.0	-951.9	-360.1	-155.0	-74.2
0.70	-2300.8	-731.8	-280.7	-122.9	-60.1
0.80	-1567.9	-499.7	-192.2	-84.1	-41.1
0.90	-771.6	-246.5	-95.1	-41.9	-20.6
1.00	0.0	0.0	0.0	0.0	0.0

[a] Accuracy of ± 0.01 K for temperature, and for relative viscosity ± 0.35 %. (0.95 level of confidence).

Table S5. Adjustment parameters of Equation 2 for Viscosity Deviation (Δη) results[a]

Parameters	T (K)				
	303.15	313.15	323.15	333.15	343.15
$\mathbf{A_0/10^{-3}}$	-13.4612	-4.1906	-1.5640	-0.6627	-0.3121
$\mathbf{A_1/10^{-4}}$	-11.8620	-3.4167	-1.2266	-0.5104	-0.2399
$\mathbf{A_2/10^{-4}}$	6.9310	2.0075	0.7285	0.3075	0.1468
$\mathbf{A_3/10^{4}}$	4.1971	3.0177	3.3429	3.0996	4.3316
$\mathbf{A_4/10^{-2}}$	-2.4756	-3.4753	-3.1762	-3.4730	-2.5185
AARD	0.088	0.083	0.080	0.079	0.079
